# The Impact of Major and Trace Elements in Serum and Bone on Dual-Energy X-Ray Absorptiometry-Derived Hip Strength

**DOI:** 10.1007/s00223-022-00945-5

**Published:** 2022-01-24

**Authors:** Tomasz Miazgowski, Aleksandra Rył, Aleksandra Szylińska, Iwona Rotter

**Affiliations:** 1 Department of Propedeutics of Internal Medicine and Arterial Hypertension, Pomeranian Medical University in Szczecin, Szczecin, Poland; 2 Department of Medical Rehabilitation and Clinical Physiotherapy, Pomeranian Medical University in Szczecin, Szczecin, Poland

**Keywords:** Hip strength, Elemental analysis, Bone mineral density, Bone health

## Abstract

The purpose of this study was to establish associations between both serum levels and bone content of a wide range of elements (Na, K, P, Ca, Mg, Zn, Cu, Cr, Mn, Fe, and Pb), with hip strength (HS) indices derived from dual-energy X-ray absorptiometry (DXA). The study population consisted of a number of male patients aged 56–77 years following hip replacement due to osteoarthritis of the hip. Bone specimens were taken from the femoral head and neck during arthroplasty. The elemental analyses were carried out using coupled plasma optical emission spectrometry. The following DXA-HS parameters were assessed: buckling ratio (BR), cross-sectional area (CSA) and its moment of inertia (CSMI), section modulus, and Femoral Strength Index (FSI). Age was positively correlated with Na, K, and Cu in the bone. Ca in the bone was positively associated with BR and negatively with SM and CSMI. Of all the DXA-HS parameters, the weakest associations of elements in the bone were found with FSI and the strongest with BR. Among the elements in the serum, the strongest negative associations were found for K, Cr, Mn, and Zn with CSA, while the majority of bone elements were associated either positively (Ca, P, Mg, Zn, and Cu) or negatively (Mn, Fe, Pb, and Cr) with BR. In conclusion, the interactions between individual elements in blood serum and bone with DXA-HS could not be unequivocally established.

## Introduction

The load-bearing capacity of bone depends on its mass, commonly assessed by densitometry and bone quality to encompasses geometry (macroarchitecture), microarchitecture, intrinsic properties of the bone tissue, and the interactions between its elements. While bone strength is determined by genetic factors, throughout its lifespan bone tissue is able to adjust its geometrical rearrangements to the mechanical loads and strains, in addition to bone homeostasis driven by the paracrine and systemic hormonal milieu [1–3]. Through these mechanisms, bone can modify its size (diameter and thickness), shape, and architecture (i.e., the redistribution of bone tissue) to adapt the cross-sectional area (CSA) and moment of inertia (CSMI) to the stresses [3–6]. The majority of these attributes arise from the specific composition of bone matrix [7]. Among the minerals involved in these bone processes, calcium (Ca) and phosphorus (P) ions (the main constituents of hydroxyapatite crystal) play a pivotal role in the acquisition and maintenance of bone strength. However, bone matrix also contains amounts of other major elements such as sodium (Na), potassium (K), and magnesium (Mg), as well as trace elements such as zinc (Zn), copper (Cu), lead (Pb), manganese (Mn), chrome (Cr), and iron (Fe) that potentially affect bone strength. It has been suggested that some of these mechanisms might be mediated through their impact on bone mass and remodeling. For example, even mild Na deficiency leads to increased osteoclast activity and bone resorption [8, 9]. One of the most promoted hypotheses for the benefit of K on bone homeostasis is through its effect on acid–base balance, as bone tissue turnover may increase in response to acid [10]. Zn, which is an important cofactor in metalloenzymes, may stimulate cell differentiation and proliferation and bone mineralization through gene expression of various proteins, including type I collagen, alkaline phosphatase, and osteocalcin [11–14]. In turn, Cu—an enzymatic cofactor—may activate lysyl oxidase, which induces the formation of lysine crosslinks in collagen and elastin and inhibits osteoclastic resorption [15]. However, in clinical studies, the associations of Cu with bone mass have yielded conflicting conclusions, with positive [16], negative [17], and neutral [18]. Other trace elements may exert different effects on bone health: negative (Pb, Al, Co, Cd, and Cr), positive (Si, B, Sr, and Mg), or antagonistic (positive or negative) depending on their intra-osseous content (Cu, Li, F, and Mn) [13, 19, 20]. Nonetheless, the relationship between the mineral composition of bone and its mechanical properties is poorly understood. Specifically, there have been no previous reports evaluating the role of the chemical elements present in the inorganic matrix in determining bone strength.

The purpose of this study was to establish associations of the serum levels and bone content of a wide range of elements (Na, K, P, Ca, Mg, Zn, Cu, Cr, Mn, Fe, and Pb) with the dual-energy X-ray absorptiometry (DXA)-derived indices of hip strength (HS).

## Material and Methods

### Study Population

The study population consisted of male patients treated at the Orthopedic Surgery Clinic of the Pomeranian Medical University due to osteoarthritis (OA) of the hip, using a routine hip replacement. We excluded patients with diabetes requiring insulin therapy, history of cancer within the preceding 5 years, liver or kidney failure, heart failure (class III or IV according to the New York Heart Association (NYHA) classification), and medications that might have potentially affected bone metabolism, such as mineral supplements, neuroleptics, chemotherapeutic agents, immunosuppressants, steroids, and antidepressants. Overall, we included 57 patients, four of whom had undergone a prior hip replacement on the opposite side. All the patients had severe OA (grade 4 according to standard Kellgren–Lawrence classification). On the opposite hip, the severity of OA was evaluated as mild to moderate (grade 2 or 3). The study complied with all applicable institutional regulations regarding the ethical use of human volunteers in research and the terms of the Declaration of Helsinki. The Pomeranian Medical University Ethics Committee approved the study protocol, and all participants gave their written consent.

### Elemental Analysis

Serum concentrations and bone content of Na, K, P, Ca, Mg, Zn, Cu, Cr, Mn, Fe, and Pb were measured using inductively coupled plasma optical emission spectrometry (ICP-OES; iCAP 7400 Duo analyser equipped with a polypropylene cyclonic spray chamber; Thermo Fisher Scientific, Waltham, MA, USA), a well-established and powerful technique commonly used for quantification of elements in liquid and solid samples. All venous blood samples were collected following an overnight fast and stored at − 80 °C until processed. The samples were thawed to room temperature and digested using the microwave digestion system CEM MARS 5. Next, samples were transferred to polypropylene tubes and 4 mL of high-purity 65% HNO_3_ reagent (Suprapur, Merck, Darmstadt, Germany) was added. After completion of the pre-reaction time, 1 mL of non-stabilized 30% H_2_O_2_ solution (Suprapur, Merck, Darmstadt, Germany) was added to each vial. Once the addition of all reagents was complete, the samples were placed in special Teflon vessels and heated in a microwaved digestion system for 35 min at 180 °C (15 min ramp up to 180 °C and maintained at 180 °C for 20 min). In a clean hood, samples were transferred to acid-washed 15-mL polypropylene sample tubes. A further fivefold dilution was performed prior to ICP-OES measurement and 2 mL was taken from each digest. The samples were then spiked with an internal standard to provide a final concentration of 0.5 mg/L Yttrium and 1 mL of 1% Triton (Triton X-100, Sigma) and diluted to a final volume of 10 mL with 0.075% nitric acid (Suprapur, Merck, Germany). Blank samples were prepared by adding concentrated nitric acid to the tubes without sample and subsequently diluted in the same manner as described above. The calibration standards (ICP multi-element standard solution IV; AccuStandard Inc., New Haven, CT, USA) and the reference material (National Institute of Standards and Technology (NIST) SRM 8414 Bovine Muscle, Gaithersburg, MD, USA) were prepared in the same manner as the samples and blanks. The limits of detection (µg/L) were as follows: Ca 0.003, Mn 0.07, K 0.6, Zn 0.19, Cu 0.39, Fe 0.25, Na 0.37, Pb 1.06, P 1.55, and Mg 0.01.

Bone specimens were taken from the femoral head and neck following the hip arthroplasty procedure. During surgery, the soft tissues surrounding the femoral head were discarded and then the femoral head and neck were extracted from the acetabulum manually. This test material was collected and stored at − 80 °C until analysis. At analysis, samples were thawed to room temperature, dried overnight to a constant weight at 80 °C after cleaning of all adherent tissues, ground into a powder in a porcelain mortar, and mineralized using a CEM MARS 5 digestion oven. All pre-analytical procedures were similar to the serum samples. Blank samples were prepared by adding concentrated nitric acid (80 µL) to tubes without sample and subsequently diluted in the same manner. Bulk mineral calibration standards (ICP multi-element standard solution IV Ca, Mn, K, Zn, Cu, Fe, Na, Pb, Cr, P, Mg; Merck, Germany; Single Element ICP Standard for K, Inorganic Ventures, Christiansburg, VA, USA) were prepared at different concentrations of inorganic elements. Samples of reference material (NIST SRM 1486 Bone Meal) were prepared in the same manner as the bone samples.

Analyses were performed in both radial and axial mode depending on the element and matrix. Multiple wavelengths were generally monitored for each element to provide confirmation of quantitative results. Continuing calibration check samples were analyzed at most after every tenth sample and consisted of a blank measurement to monitor carryover and mid-range calibration standards from both the low and high concentration calibration curves. The wavelengths (nm) were as follows: Ca 315.887, Mn 257.610, K 766.490, Zn 206.200, Cu 224.700, Fe 238.204, Na 589.592, Pb 220.353, P 178.284, and Mg 280.270.

### Bone Mineral Density

BMD was measured by DXA (Lunar Prodigy Advance, enCORE software version 14.10; GE Healthcare; Madison, WI, USA). The total body BMD and bone mineral content (BMC) were assessed using the Advanced Body Assessment tool of the software system. Total neck BMD at the left and right femur was measured without reposition using the DualFemur™ scan mode on all but four of the patients with a prior hip replacement, in which a single femur scan mode on the operated side was used.

### Hip Strength Assessment

HS was assessed using the Advanced Hip Structural Analysis (HSA) software package that derives the cross-sectional geometry from plain images acquired by DXA. This method uses raw spatial and mineral mass from the proximal femur to compute structural geometrical indices at three specific locations: the neck, and the intertrochanteric and proximal shaft regions. The software computes the following parameters: (1) CSMI (in mm^4^) that is used to measure the distribution of material around the neck axis necessary to calculate resistance to bending forces—mechanical stress within a cross-section subjected to bending is inversely related to the CSMI and varies with the distance from the neutral axis [21]; (2) CSA (in mm^2^), the total surface area of bone in a cross-sectional slice after excluding all the spaces occupied by marrow and other soft tissues within pores. CSA is commonly referred to as the minimal CSMI section within the neck ROI and reflects the ability of the femoral neck to withstand axial forces; (3) neck shaft angle, the angle between the femoral neck and femoral shaft (an axillary parameter, not evaluated in this study); (4) section modulus (SM), a strength parameter derived from CSMI, equal to the CSMI divided by the distance from centroidal axis to the edge of the section; (5) Femoral Strength Index (FSI), a composite unitless index that corresponds to a ratio of the estimated compressive yield strength of the femoral neck to the expected compressive stress of a fall on the greater trochanter, adjusted for age, height and weight. The greater the FSI, the lower the hip fracture risk from a fall on the greater trochanter; and (6) buckling ratio (BR), the ratio of the outer radius to the cortical thickness, which represents a means for estimating the stability of the cortex in thin-walled regions subjected to bending; a higher BR value means a greater instability and thus it makes sense that greater strength is found at regions with greater stability, i.e., lower BR. The HSA, similarly to BMD, was shown to be an independent predictor of fragility fractures [22, 23].

### Statistical Analysis

Descriptive statistics were presented as means (ranges) and standard deviations (SD) for continuous variables and frequency distribution for categorical variables. Data were checked for normality using a Shapiro–Wilk test. A comparison of HS and BMD between the left and right femur was assessed using a non-parametric Mann–Whitney *U*-test. Correlations between pairs of quantitative variables were analyzed using a Pearson’s linear correlation or Spearman’s rho correlation for normally and non-normally distributed variables. The associations of serum and bone element levels (predictors) with HS indices (outcomes) were assessed using multiple linear regression models adjusted for age, BMI, and femur BMD. Statistical analyses were performed using Statistica (v12.0; StatSoft Poland).

## Results

### The Study Population

The study population comprised 57 males aged 56–77 years (Table 1). Among them, 16 were obese, and 4 had a prior hip replacement of the opposite hip. 43 patients were being treated for hypertension, 22 had benign prostatic hyperplasia, 8 had type 2 diabetes, and 4 had hyperuricemia. Two patients (3.5%) met the DXA diagnostic criteria for osteoporosis (femur *t*-score ≤ 2.5 SD) and 6 (10.5%) had mildly reduced BMD (femur *t*-score between − 1 and − 2 SDs). None of the patients had a history of previous hip fracture.

**Table 1:** Baseline characteristics of study participants (*n* = 57)

	Mean	SD	Range
Age [years]	66.70	4.967	56.0–77.0
Height [cm]	175.1	15.22	165–193
Weight [kg]	89.87	7.824	73.0–112.5
Body mass index [kg/m^2^]	30.03	3.392	22.77–37.88
Current smokers (*n*; %)	6 (10.5%)		
Hypertension, *n* (%)	43 (75.4%)		
Diabetes, *n* (%)	8 (14.0%)		
Hyperuricemia, *n* (%)	4 (7.0%)		
Benign prostatic hyperplasia, *n* (%)	22 (38.6%)		
Obesity, *n* (%)	16 (28.1%)		
Operated femur (left/right)	29 (50.9%)/28 (49.1%)		
Total bone mineral density [g/cm^2^]	1.270	0.155	0.991–1.555
Bone mineral content [kg]	3.089	0.403	2.086–4.012

The levels of the elements in the serum and bone are presented in Tables 2 and 3, respectively. The mean values of elements in the serum were within ICP-OES reference ranges in literature [24, 25]. The levels of the elements in the bone were in the following descending order: Ca (62.4% of total elements) > P (34.1%) > Mg > K > Zn > Fe > Pb > Cr > Cu > Mn. The mean values of femur BMD, *t*-score, *z*-score, and all HS parameters (calculated for 53 patients who had no prior hip replacement) did not differ significantly in comparison between the operated and non-operated side (*p* > 0.1 for all comparisons). As expected, femur BMD was significantly correlated with all strength indices. The strongest associations were with CSA (*r* = 0.853; *p* = 0.001) followed by SM (*r* = 0.635; *p* = 0.001), CSMI (*r* = 0.473; *p* = 0.001), BR (*r* =  − 0.305; *p* = 0.033), and FSI (*r* = 0.301; *p* = 0.035).

**Table 2:** Concentration of serum elements

	Mean	SD	Range	Reference range^a^
Na [mg/L]	3395	453.6	2518–4760	3103–3402
K [mg/L]	204.4	51.09	130–350	149–215
Ca [mg/L]	112.8	6.660	86–147	91–106
P [mg/L]	219.3	59.38	130–342	85.9–246
Zn [mg/L]	1.395	0.368	0.84–2.35	0.70–1.20
Cu [mg/L]	0.992	0.150	0.69–1.46	0.80–1.50
Fe [mg/L]	1.500	0.389	0.80–2.51	0.70–1.500
Cr [mg/L]	0.008	0.002	0.003–0.01	0.001–0.041
Mg [mg/L]	25.80	3.742	16.9–38.2	17.0–22.0
Mn [mg/L]	0.009	0.003	0.006–0.02	0.005–0.018
Pb [μg/L]	0.724	0.099	0.62–0.82	0.033–6.325

^a^Reference ranges from ref [24, 25]

**Table 3:** Bone elements

	Mean	SD	Range
Na [g/kg]	9.359	3.027	3.130–13.94
K [mg/kg]	839.7	389.1	71.25–1581.8
Ca [g/kg]	262.6	80.49	87.54–392.8
P [g/kg]	143.6	42.95	64.74–262.0
Zn [mg/kg]	195.7	53.34	68.08–306.4
Cu [mg/kg]	1.439	0.898	0.624–4.185
Fe [mg/kg]	162.9	243.8	0.585–1123
Cr [mg/kg]	1.669	2.813	0.242–10.91
Mg [g/kg]	3.538	1.001	1.220–5896
Mn [mg/kg]	0.760	1.253	0.101–5.130
Pb [mg/kg]	2.797	1.397	0.788–5.718

### Associations of Elements in the Serum with Bone Mass and Hip Strength

Serum Ca concentration did not correlate with either BMD or HS (Table 4). A similar lack of correlation was also found for the other major elements, apart from serum P level, which was weakly but significantly associated with FSI. From among the trace elements, Fe level was correlated with CSA, BMD, and BMC, and Mn level with BMD and BMC. However, after adjusting for age, BMI, and femur BMD, these associations were no longer significant (Table 5). Instead, serum Cr, Mn, K, and Zn were found to be significant, negatively correlated determinants of HS parameters. In contrast, serum P concentration was positively associated with HS (but only in relation to CSMI).

**Table 4:** Correlations of serum elements with bone indices

	FSI	Buckling ratio	Section modulus	CSMI	CSA	Femur BMD	Total BMD	Total BMC
Na	− 0.050	− 0.167	− 0.082	− 0.153	0.023	0.176	0.211	0.225
K	− 0.292	0.224	− 0.248	− 0.223	− 0.082	0.209	0.221	0.155
Ca	0.176	− 0.222	0.023	0.001	0.191	0.216	0.312	0.301
P	0.358^a^	0.176	0.222	0.260	0.045	− 0.117	− 0.152	− 0.127
Zn	− 0.329	0.120	− 0.189	− 0.239	− 0.036	0.234	0.301	0.209
Cu	− 0.253	− 0.133	− 0.137	− 0.160	− 0.177	− 0.108	− 0.083	− 0.109
Fe	0.125	− 0.334	0.280	0.280	0.442^b^	0.410^a^	0.524^b^	0.557^b^
Cr	0.020	0.130	− 0.066	− 0.081	− 0.082	0.065	0.048	− 0.022
Mg	− 0.223	0.136	− 0.232	− 0.271	− 0.083	0.155	0.183	0.121
Mn	− 0.198	0.223	− 0.006	− 0.063	0.127	0.333^a^	0.419^a^	0.298
Pb	− 0.892	0.117	− 0.192	− 0.094	− 0.114	− 0.109	− 0.202	− 0.198

^a^*p* < 0.05; ^b^*p* < 0.01

**Table 5:** Associations of serum elements with HS

	Hip strength index	Adjusted for age, BMI and femur BMD			
		Beta	*p*-value	95% CI	
Zn	Section modulus	− 0.397	0.028	− 0.750	− 0.044
	CSA	− 0.799	0.001	− 1.240	− 0.358
Mn	Section modulus	− 0.568	0.001	− 0.906	− 0.230
	CSMI	− 0.404	0.011	− 0.713	− 0.096
	CSA	− 0.806	0.001	− 1.255	− 0.357
Cr	Section modulus	− 0.561	0.003	− 0.915	− 0.208
	CSMI	− 0.413	0.013	− 0.734	− 0.093
	CSA	− 0.810	0.001	− 1.279	− 0.342
K	Buckling ratio	0.316	0.034	0.026	0.607
	Section modulus	− 0.573	0.002	− 0.916	− 0.230
	CSMI	− 0.381	0.021	− 0.702	− 0.060
	CSA	− 0.842	0.001	− 1.295	− 0.389
P	CSMI	0.323	0.031	0.031	0.614

In comparison with serum and bone levels, Na and Cu were significantly correlated (*r* = 0.421; *p* = 0.007 and *r* = 0.363; *p* = 0.021, respectively), while the other elements were not. After adjusting for age, smoking, and total BMD, serum and bone levels of Na and K were positively associated, and for Fe levels, the association was negative (Table 6).

**Table 6:** Serum vs. bone element concentration adjusted for age, smoking and total BMD

	Beta	95% CI		*p*-value
Ca	0.067	− 0.261	0.396	0.680
P	− 0.057	− 0.057	− 0.344	0.692
Na	0.168	0.086	0.765	0.015
K	0.434	0.049	0.819	0.028
Zn	− 0.034	− 0.034	− 0.360	0.836
Cu	0.205	− 0.101	0.511	0.183
Fe	− 0.573	− 0.872	− 0.273	0.001
Cr	− 0.255	− 0.601	0.090	0.143
Mg	− 0.005	− 0.346	0.337	0.979
Mn	− 0.150	− 0.530	0.231	0.431
Pb	0.101	0.026	0.142	0.058

### Associations of Elements in the Bone with Bone Mass and Hip Strength

Age was positively correlated only with bone content of Na (*r* = 0.312; *p* = 0.047), K (*r* = 0.619; *p* = 0.001), and Cu (*r* = 0.395; *p* = 0.011). BMI was correlated with Mn (*r* = 0.442; *p* = 0.004) and Fe content (*r* = 0.447; *p* = 0.003). No other significant interactions of age and BMI with elements in bone were observed.

As shown in Table 7, Na content in bone was negatively correlated with BR and positively with BMC. Other significant associations included a negative correlation of K with FSI; Ca negatively with BR and positively with BMD and BMC; P positively with FSI and CSMI and negatively with FSI, BR, SM, and CSMI; Zn negatively with BR; Cu and Cr negatively with FSI; Mg negatively with BR and positively with BMD and BMC; and Mn negatively with BR. Of all the studied elements, only bone Fe and Pb were not correlated with BMD and HS parameters. However, after adjustment for age and BMI (Table 8), Ca, P, Mg, Na, and Zn were inversely associated with BR (a positive effect on buckling resistance) with the strongest effects of P and Zn (β <  − 0.5). Mn, Fe, Pb , and Cr were associated with a negative effect on buckling strength. Of other HS parameters, CSMI was negatively associated with bone P and K, and the FSI with Cu and Cr (a negative relationship). Importantly, most relationships between bone elements and HS remained significant after further adjustment for BMD, particularly those with BR. In addition, Ca content showed an ambivalent relationship with HS parameters: either positive (in relation to buckling resistance) or negative (to SM and CSMI). A similar pattern was followed by P, Zn, and Mg. The remaining major elements (Na and K) were associated only with SM and CSMI. Generally, of all DXA-HSA parameters, the weakest associations of the elements in bone were found to be with FSI and the strongest with BR.

**Table 7:** Correlations of bone elements with bone strength indices and BMD

	FSI	Buckling ratio	Section modulus	CSMI	CSA	Femur BMD	Total BMD	Total BMC
Na	− 0.197	− 0.461^b^	0.007	− 0.081	0.141	0.302	0.315	0.353^a^
K	− 0.345^a^	− 0.236	− 0.173	− 0.219	− 0.186	− 0.088	− 0.080	− 0.088
Ca	− 0.136	− 0.444^b^	0.035	− 0.043	0.190	0.382^a^	0.364^a^	0.424^b^
P	0.371^a^	− 0.432 ^a^	− 0.359^a^	− 0.414^a^	− 0.292	− 0.001	0.170	0.077
Zn	− 0.235	− 0.500^a^	− 0.053	− 0.121	0.098	0.282	0.241	0.314
Cu	− 0.420^a^	− 0.278	− 0.151	− 0.267	− 0.245	− 0.224	− 0.153	− 0.175
Fe	− 0.134	0.260	0.049	0.059	− 0.059	− 0.004	− 0.002	− 0.001
Cr	− 0.383^a^	− 0.312	− 0.181	− 0.292	− 0.283	− 0.305	− 0.263	− 0.255
Mg	− 0.187	− 0.465^a^	− 0.019	− 0.100	0.145	0.343^a^	0.329	0.393^a^
Mn	− 0.111	0.345^a^	0.007	0.025	− 0.049	− 0.040	− 0.041	0.049
Pb	− 0.059	− 0.246	− 0.068	− 0.145	− 0.016	0.100	0.046	0.098

^a^ p < 0.05; ^b^ p < 0.01

**Table 8:** Associations of bone elements with HS

	Hip strength index	Adjusted for age and BMI				Adjusted for age, BMI and BMD			
		Beta	*p*	95% CI		Beta	*p*	95% CI	
Ca	FSI	− 0.106	0.512	− 0.431	0.218	− 0.203	0.207	− 0.523	0.117
	Buckling ratio	− **0.439**	0.003	− 0.719	− 0.158	** − 0.384**	0.021	− 0.705	− 0.062
	SM	− 0.133	0.445	− 0.481	0.215	** − 0.497**	0.013	− 0.881	− 0.113
	CSMI	− 0.239	0.157	− 0.574	0.096	** − 0.463**	0.008	− 0.796	− 0.129
	CSA	0.109	0.541	− 0.249	0.467	− 0.407	0.136	− 0.948	0.135
P	FSI	− 0.170	0.303	− 0.501	0.160	− 0.153	0.378	− 0.499	0.194
	Buckling ratio	** − 0.513**	0.001	− 0.790	− 0.237	** − 0.728**	0.001	− 1.011	− 0.446
	SM	− 0.241	0.174	− 0.592	0.111	− 0.263	0.232	− 0.702	0.175
	CSMI	** − 0.404**	0.017	− 0.731	− 0.077	** − 0.435**	0.021	− 0.801	− 0.069
	CSA	− 0.185	0.311	− 0.549	0.179	− 0.239	0.420	− 0.831	0.354
Mg	FSI	− 0.147	0.357	− 0.468	0.173	− 0.225	0.167	− 0.548	0.098
	Buckling ratio	** − 0.472**	0.001	− 0.744	− 0.200	** − 0.474**	0.004	− 0.788	− 0.160
	SM	− 0.203	0.238	− 0.544	0.139	** − 0.527**	0.009	− 0.913	− 0.141
	CSMI	− 0.322	0.052	− 0.646	0.002	** − 0.521**	0.003	− 0.851	− 0.190
	CSA	0.030	0.868	− 0.327	0.386	− 0.436	0.115	− 0.983	0.111
K	FSI	− 0.226	0.100	− 0.496	0.045	− 0.227	0.115	− 0.511	0.058
	Buckling ratio	− 0.071	0.594	− 0.341	0.198	− 0.126	0.413	− 0.434	0.182
	SM	** − 0.381**	0.008	− 0.656	− 0.106	** − 0.525**	0.003	− 0.859	− 0.191
	CSMI	** − 0.383**	0.006	− 0.651	− 0.115	** − 0.438**	0.005	− 0.735	− 0.140
	CSA	− 0.220	0.147	− 0.521	0.081	− 0.443	0.070	− 0.923	0.038
Na	FSI	− 0.135	0.397	− 0.453	0.183	− 0.229	0.147	− 0.543	0.084
	Buckling ratio	** − 0.356**	0.016	− 0.643	− 0.069	− 0.286	0.085	− 0.614	0.041
	SM	− 0.169	0.322	− 0.510	0.172	** − 0.537**	0.006	− 0.909	− 0.165
	CSMI	− 0.255	0.124	− 0.582	0.073	** − 0.474**	0.006	− 0.800	− 0.147
	CSA	0.095	0.590	− 0.258	0.447	− 0.410	0.128	− 0.944	0.123
Mn	FSI	− 0.144	0.349	− 0.450	0.163	− 0.078	0.619	− 0.394	0.238
	Buckling ratio	**0.390**	0.006	0.120	0.659	**0.357**	0.025	0.047	0.668
	SM	− 0.257	0.134	− 0.597	0.083	− 0.128	0.571	− 0.583	0.326
	CSMI	− 0.144	0.389	− 0.480	0.191	− 0.027	0.885	− 0.399	0.346
	CSA	− 0.284	0.096	− 0.619	0.052	− 0.168	0.582	− 0.781	0.445
Zn	FSI	− 0.145	0.372	− 0.469	0.179	− 0.220	0.182	− 0.549	0.108
	Buckling ratio	** − 0.519**	0.001	− 0.786	− 0.251	** − 0.537**	0.001	− 0.846	− 0.229
	SM	− 0.171	0.325	− 0.519	0.177	** − 0.475**	0.021	− 0.874	− 0.075
	CSMI	− 0.290	0.084	− 0.622	0.041	** − 0.479**	0.007	− 0.822	− 0.137
	CSA	0.060	0.739	− 0.301	0.420	− 0.347	0.219	− 0.909	0.215
Cu	FSI	** − 0.298**	0.049	− 0.594	− 0.002	− 0.281	0.075	− 0.591	0.030
	Buckling ratio	− 0.221	0.134	− 0.512	0.071	** − 0.368**	0.025	− 0.689	− 0.048
	SM	− 0.112	0.502	− 0.444	0.221	− 0.037	0.856	− 0.451	0.376
	CSMI	− 0.231	0.150	− 0.550	0.088	− 0.207	0.249	− 0.564	0.150
	CSA	− 0.155	0.362	− 0.494	0.185	− 0.091	0.741	− 0.643	0.461
Fe	FSI	− 0.151	0.302	− 0.444	0.141	− 0.110	0.467	− 0.414	0.193
	Buckling ratio	**0.327**	0.017	0.061	0.592	**0.314**	0.045	0.008	0.620
	SM	− 0.220	0.161	− 0.531	0.091	− 0.159	0.412	− 0.545	0.228
	CSMI	− 0.136	0.377	− 0.445	0.173	− 0.066	0.699	− 0.408	0.276
	CSA	− 0.220	0.170	− 0.539	0.099	− 0.160	0.536	− 0.679	0.359
Pb	FSI	− 0.036	0.825	− 0.359	0.288	− 0.092	0.622	− 0.213	0.196
	Buckling ratio	**0.339**	0.024	0.631	0.047	**0.334**	0.042	0.629	0.056
	SM	− 0.218	0.204	− 0.558	0.123	− 0.040	0.792	− 0.866	0.445
	CSMI	− 0.312	0.059	− 0.638	0.013	− 0.116	0.274	− 0.496	0.293
	CSA	− 0.153	0.387	− 0.506	0.200	− 0.199	0.325	− 0.450	0.411
Cr	FSI	** − 0.306**	0.049	− 0.609	− 0.002	− 0.234	0.075	− 0.631	0.033
	Buckling ratio	**0.304**	0.040	− 0.594	− 0.014	**0.491**	0.003	− 0.801	− 0.181
	SM	− 0.122	0.483	− 0.472	0.227	− 0.032	0.781	− 0.466	0.402
	CSMI	− 0.251	0.137	− 0.585	0.083	− 0.219	0.241	− 0.591	0.153
	CSA	− 0.167	0.343	− 0.518	0.184	** − **0.078	0.781	− 0.645	0.489

Significant associations are bolded

*SM* section modulus, *CSMI* cross-sectional moment of inertia, *CSA* cross-sectional area

The mean value of the Ca/P ratio in bone was 1.908 ± 0.21 and was not associated with any of the HS indices after adjusting for age, BMI, and BMD. When BMD was removed from the regression model, the associations remained insignificant.

## Discussion

The vast majority of existing studies assessed the role of elements in maintaining bone health by using serum concentrations in relation to BMD, bone turnover, or susceptibility to fractures. This is the first study that provides new insights into in vivo bone strength using a comprehensive analysis of both serum and bone levels of elements in relation to DXA-HS. We found that although BMD was strongly correlated with CSA and SM, its relation with other HS indices, especially BR and FSI, was relatively weaker suggesting that aside from BMD other factors may contribute to bone strength. Indeed, although BMD is a good indicator of bone mass and resistance to external and internal forces, in quantitative terms, BMD is predominantly a measure of the mineral compound calcium hydroxyapatite [1, 21, 22], which has the formula Ca_10_ (PO_4_)_6_ (OH)_2_; however, extracellular inorganic bone matrix also contains other non-phosphate calcium compounds (e.g., calcium carbonate) as well as other chemical elements that are not measurable by conventional DXA [1, 22]. Our study demonstrated a lack of association of serum calcium with the BMD (both total and femur), BMC, and HS indices. By contrast, bone Ca content was positively associated with BMD, BMC, and HS, and although the association with HS was limited solely to buckling resistance, it remained significant even after controlling for confounders. This may indicate that most of the strengthening effects of bone Ca are driven by hydroxyapatite density and are limited to withstanding buckling rather than compressive and axial forces applied to the femur. Moreover, we found that serum and bone Ca levels were not correlated. Taken together, these findings suggest that in males over 55, bone-bound Ca is regulated by local mechanisms, which likely are independent of serum concentrations, at least in subjects without overt osteoporosis and normal Ca serum levels. If so, a high Ca ingestion (unintended or in supplements) would not necessarily improve bone health. This suggestion is consistent with an earlier meta-analysis reporting the poor efficacy of Ca supplementation in older males [26].

Phosphorus, another main component of hydroxyapatite crystals, when measured in serum and bone, displayed ambiguous associations with HS. Serum P was positively associated with CSMI but not with BMD or other HS parameters. The bone content of P was negatively associated with CSMI, and also with BR after corrections for confounders. An interpretation of these findings seems uncertain. It has been suggested that the Ca/P ratio in bone compared to each element separately might be a better indicator of variation in BMD as it can be driven by changes in either Ca or P individually or dissimilar changes in both [27, 28]. However, our results indicate that this suggestion does not apply to HS. Therefore, further studies are needed to establish the role of the Ca/P ratio in determining bone strength.

Aside from Ca and P, other major elements were also found to be associated with HS. Potassium is a ubiquitous element, but its role in bone homeostasis remains unclear. It was shown that supplementation with potassium citrate may enhance Ca absorption as well as decrease urinary Ca excretion, bone resorption markers, and serum PTH level [20, 29, 30]. On the other hand, several studies could not demonstrate any beneficial effects of long-term potassium citrate supplementation on bone turnover and BMD [31], supporting a suggestion that bone metabolism seems to be relatively insensitive to imbalances in total body potassium [20]. Nevertheless, our study demonstrated negative associations of both serum and bone K with CSMI, SM, and buckling strength, suggesting a detrimental effect of potassium on HS. A similar role can be attributed to sodium and magnesium. Physiologically, sodium is the extracellular counterpart of K. It is generally believed that bone disease is not associated with Na deficiency or excess [9, 20], although some studies have demonstrated the impact of hyponatremia on the risk of osteoporosis in rats and older humans [32]. In our study, under normal serum level, sodium in bone was inversely associated with BR, SM, and CSMI. Whether or not these associations play any role in a hyponatremic state remains unknown and warrants future studies. Magnesium, which is an integral component of the apatite crystals, supports the production of hydroxyapatite, bone marrow stromal cells mineralization, and 1,25(OH)2D vitamin synthesis [33]. We found serum Mg not to be associated with any of the bone indices. In contrast, bone Mg was positively correlated with BMC and femur BMD and negatively with BR. The relationship with BR remained significant after correcting for confounders. However, bone Mg was negatively associated with SM and CSMI; therefore, the net effect of Mg on HS seems ambiguous.

Among the trace elements, manganese plays numerous roles as a cofactor in the formation of bone collagen as well as bone mineralization [13], but on the other hand, Mn overload can impair bone development, in addition to neurotoxicity, its major side effect [20]. We found Mn not to be associated with BMD and BMC. In contrast, the element was negatively associated with HS regardless of age, BMI, and femur BMD. These effects were observed for Mn both in serum (in relation to SM, CSMI, and CSA) and bone (solely to BR). Therefore, despite both the acute toxicity and chronic neurotoxicity of Mn resulting from high daily intake in humans being rare [20], our results suggest that the accumulation of Mn in bone might be deleterious to bone strength. Another trace mineral, chromium, is known from animal models and in vitro studies to induce oxidative stress and cytotoxic effects on bone cells leading to accelerated bone resorption [34, 35]. Cr-related acute intoxication in humans is rare from environmental exposure; however, in long-term exposure, Cr accumulation can lead to a higher susceptibility to fractures [20] and, as suggested by this study, possibly also a reduction in bone strength, especially in response to buckling forces. Zn behaved similarly to Mg on HS: Zn bone content was significantly positively associated with BR and negatively with SM and CSMI, and in contrast to Mg, serum Zn was strongly negatively associated with CSA. Interestingly, although earlier studies evaluating associations of Zn with BMD yielded inconsistent conclusions [18, 19, 36], our results indicate that at least some of the effects of Zn on HS could be mediated independently of BMD. Similar discrepancies exist for Cu in determining BMD and fracture risk [13–18]. In this study, serum Cu was poorly correlated with HS, while bone Cu was negatively associated with FSI, and after adjusting for BMD, Cu was positively associated with buckling strength.

In the remaining trace elements, bone Fe and Pb were inversely associated with BR. In addition, serum Fe correlated positively with BMD (both femur and total), BMC, and CSA; however, after correcting for confounders, the associations were no longer significant. Iron in normal concentrations regulates bone turnover. However, the beneficial Fe concentration window is relatively narrow and Fe overload may intensify bone resorption and oxidative stress, as well as reduce bone biomechanical properties [20, 33, 37]. Lead, in turn, easily accumulates in bones even at a low level of exposure and is believed to be highly cytotoxic to the bone tissue, affecting osteoblasts, osteoclasts, and chondrocytes [20, 38]. Importantly, in our study, both elements were noticeably associated with diminished femur strength, despite their serum levels being normal.

In this study, serum and bone analyses of all the elements were performed after adjustments for age, BMI, and BMD. Such adjustments are significant as the concentrations of some elements depended on age (Na, K, and Cu) and BMI (Fe and Mn), as well as on BMD and DXA-HS indices in similar studies [22, 23].

This study had some limitations. Firstly, a major limitation of HSA with DXA is the two-dimensional nature of DXA. This method is incapable of measuring material volume, composition, or structural design of cortical and trabecular bone, as well as the muscular and genetic contributions to bone strength. Hence, areal BMD may explain only 50–70% of variation in bone strength [3]. In addition, HSA is highly sensitive to positioning, and even small changes in femur rotation have a large effect on the projected dimensions from which the femur geometry is measured [22]. To minimize these uncertainties in this study, all scans and analyses were verified by a single trained technician. Secondly, bone samples were treated with solvents in order to remove collagen, fat, and marrow before the studied elements were quantified using ICP-OES. It has been suggested that some elements are lost by this process and their content in bone might be also affected [39]. Thirdly, apart from the Ca/P ratio, all elements were evaluated separately. In the mineralized extracellular matrix, they may be present as part of different chemical compounds and in different ratios. In this context, elements may exert either beneficial or detrimental effects on bone homeostasis depending on their concentration as well as their interactions between individual elements [33]. Fourthly, due to limitations in the methods used in this study, we did not assess the water content of the bone, which is an integral constituent that influences the mechanical properties of bone. Approximately 20% of the cortical bone consists of water, which is bound to the collagenous structure, embedded in the crystal lattice and freely residing in the network of pores, playing an important role in matrix mineralization and overall bone resistance [40, 41]. Finally, bone samples were obtained from patients with hip OA, and hence, our findings may not apply to healthy individuals or patients with other bone disorders, as hip OA is associated with chronic inflammatory state that involves auricular cartilage, subchondral cortical and trabecular bone, and synovium, leading to articular cartilage stress distribution changes with subchondral bone expansion. Although in the studied cohort OA lesions were bilateral (but more severe on the operated side), bone parameters were comparable between the operated and intact femurs suggesting that the severity of OA had no essential impact on BMD and HS. In contrast, patients with OA frequently have higher femur BMD in comparison with controls, likely with the exception of a subset of patients with atrophic OA [42]. It is believed that higher BMD in OA may reflect a process known as buttressing, whereby osteophytes extend across the femoral neck, causing artefactual increases in bone mass measured by DXA [43]. Nonetheless, it is still unknown whether these changes in bone mass affect bone strength in OA patients.

In summary, the interactions between the individual elements in the blood serum and bone measured using DXA-HS indices could not unequivocally be established. Among the elements in the serum, the strongest negative associations with CSA were found for K, Cr, Mn, and Zn, while for buckling resistance, the majority of bone elements were associated either positively (Ca, P, Mg, Zn, and Cu) or negatively (Mn, Fe, Pb, and Cr).

## Data Availability

The data presented in this study are available on request from the corresponding author.
